# Interactions between NAD+ metabolism and immune cell infiltration in ulcerative colitis: subtype identification and development of novel diagnostic models

**DOI:** 10.3389/fimmu.2025.1479421

**Published:** 2025-02-05

**Authors:** Linglin Tian, Huiyang Gao, Tian Yao, Yuhao Chen, Linna Gao, Jingxiang Han, Lanqi Zhu, He Huang

**Affiliations:** ^1^ Department of Gastroenterology, First Hospital of Shanxi Medical University, Taiyuan, Shanxi, China; ^2^ The First Clinical Medical College, Shanxi Medical University, Taiyuan, Shanxi, China; ^3^ Department of Gastrointestinal Surgery, First Hospital of Shanxi Medical University, Taiyuan, Shanxi, China; ^4^ Center of Clinical Epidemiology and Evidence Based Medicine, Shanxi Medical University, Taiyuan, Shanxi, China; ^5^ Department of Nutrition and Food Hygiene, School of Public Health, Shanxi Medical University, Taiyuan, Shanxi, China

**Keywords:** ulcerative colitis, NAD+ metabolism, bioinformatics, machine learning, immune cell infiltration, subtype, diagnosis

## Abstract

**Background:**

Ulcerative colitis (UC) is a chronic inflammatory disease of the colonic mucosa with increasing incidence worldwide. Growing evidence highlights the pivotal role of nicotinamide adenine dinucleotide (NAD+) metabolism in UC pathogenesis, prompting our investigation into the subtype-specific molecular underpinnings and diagnostic potential of NAD+ metabolism-related genes (NMRGs).

**Methods:**

Transcriptome data from UC patients and healthy controls were downloaded from the GEO database, specifically GSE75214 and GSE87466. We performed unsupervised clustering based on differentially expressed NAD+ metabolism-related genes (DE-NMRGs) to classify UC cases into distinct subtypes. GSEA and GSVA identified potential biological pathways active within these subtypes, while the CIBERSORT algorithm assessed differential immune cell infiltration. Weighted gene co-expression network analysis (WGCNA) combined with differential gene expression analysis was used to pinpoint specific NMRGs in UC. Robust gene features for subtyping and diagnosis were selected using two machine learning algorithms. Nomograms were constructed and their effectiveness was evaluated using receiver operating characteristic (ROC) curves. Reverse transcription quantitative polymerase chain reaction (RT-qPCR) was conducted to verify gene expression in cell lines.

**Results:**

In our study, UC patients were classified into two subtypes based on DE-NMRGs expression levels, with Cluster A exhibiting enhanced self-repair capabilities during inflammatory responses and Cluster B showing greater inflammation and tissue damage. Through comprehensive bioinformatics analyses, we identified four key biomarkers (AOX1, NAMPT, NNMT, PTGS2) for UC subtyping, and two (NNMT, PARP9) for its diagnosis. These biomarkers are closely linked to various immune cells within the UC microenvironment, particularly NAMPT and PTGS2, which were strongly associated with neutrophil infiltration. Nomograms developed for subtyping and diagnosis demonstrated high predictive accuracy, achieving area under curve (AUC) values up to 0.989 and 0.997 in the training set and up to 0.998 and 0.988 in validation sets. RT-qPCR validation showed a significant upregulation of NNMT and PARP9 in inflamed versus normal colonic epithelia, underscoring their diagnostic relevance.

**Conclusion:**

Our study reveals two NAD+ subtypes in UC, identifying four biomarkers for subtyping and two for diagnosis. These findings could suggest potential therapeutic targets and contribute to advancing personalized treatment strategies for UC, potentially improving patient outcomes.

## 1 Introduction

UC is a chronic inflammatory bowel disease (IBD) that primarily affects the colonic mucosa, beginning in the rectum and potentially extending to the entire colon. It is clinically characterized by recurrent episodes of bloody diarrhea and abdominal pain. Globally, the incidence of UC is increasing, with an estimated five million people affected as of 2023 (1). As a chronic disease, UC significantly affects the quality of life of patients, necessitating continuous medical care and potentially leading to severe complications, including colorectal cancer (2). Despite advancements in treatments, including immunosuppressants and biologics, 10%–20% of patients suffer from recurrent and treatment-resistant symptoms, with some requiring colectomy (3). The complex interplay of environmental triggers, genetic predispositions, and immune dysregulation complicates the etiology of UC (4). At the molecular level, UC is characterized by the activation of immune cells, including T cells, macrophages, and dendritic cells, which infiltrate the colonic mucosa (5). These immune cells release pro-inflammatory cytokines, such as TNF-α, IL-1β, and IL-6, contributing to the chronic inflammation observed in UC (6). Additionally, the intestinal epithelial barrier is compromised, allowing microbial products to trigger further immune activation and inflammatory responses (7). The dysregulation of key signaling pathways, such as NF-κB and JAK/STAT, plays a crucial role in sustaining this inflammatory environment (8, 9). Furthermore, genetic factors, including mutations in immune-related genes like NOD2 and IL-23R, have been associated with an increased risk of UC (10, 11), highlighting the need for advanced research to develop more effective diagnostic and therapeutic options and to have a better understanding of its pathogenesis.

NAD+ is essential in cellular metabolism, which is critical in oxidative reactions and energy production. Beyond its metabolic functions, NAD+ is essential for maintaining cellular health, as it is involved in DNA repair, signal transduction, and cell death regulation (12). Recent studies have emphasized its importance in regulating inflammation and immune responses, which are crucial in the pathophysiology of various chronic diseases, including autoimmune and inflammatory conditions (13–15). In the context of ulcerative colitis (UC), disturbances in NAD+ metabolism are associated with the characteristics of the exacerbated inflammatory environment of the disease (16). Despite its critical role, the specific impact of NAD+ metabolism on UC is unknown (17). Therefore, investigating NAD+ metabolism in UC may lead to new therapeutic strategies that can alleviate inflammation and promote mucosal healing, providing new directions for research and treatment of the disease.

The genes identified in our study—AOX1, NAMPT, NNMT, PTGS2, and PARP9—play crucial roles in inflammation and immune cell metabolism. AOX1 is involved in the regulation of oxidative stress, a key factor in inflammatory responses (18). NAMPT plays a pivotal role in NAD+ biosynthesis, thus influencing immune cell energy metabolism and inflammatory cytokine production (19–23). NNMT has been linked to regulating methylation processes in immune cells, affecting their activation and function in inflammatory environments (24–27). PTGS2 (COX-2) is a well-known enzyme involved in the production of prostaglandins, which mediate inflammation and immune responses (28, 29). Finally, PARP9 is implicated in DNA repair and the regulation of immune cell survival, particularly in response to inflammatory stimuli (30–32). These genes contribute to the modulation of immune cell infiltration and inflammation in UC, underscoring their potential as biomarkers for both disease subtyping and diagnosis.

In this study, we collected UC samples from public databases and employed unsupervised clustering to categorize them into two NAD+ metabolism-related subtypes, clusters A and B. Our analyses revealed that these subtypes exhibited varying responses to inflammation: Cluster A exhibited improved self-repair capabilities, whereas cluster B was prone to more severe inflammation and tissue injury. Subsequently, we identified key biomarkers related to NAD+ metabolism using differential analysis, WGCNA, least absolute shrinkage and selection operator (LASSO) regression, and random forest (RF) algorithms. These biomarkers exhibited high predictive accuracy for UC subtyping and diagnosis. RT-qPCR validation of these findings offers potential new strategies and scientific bases for UC diagnosis and personalized treatment.

## 2 Materials and methods

### 2.1 Data acquisition and processing

**Figure 1:**
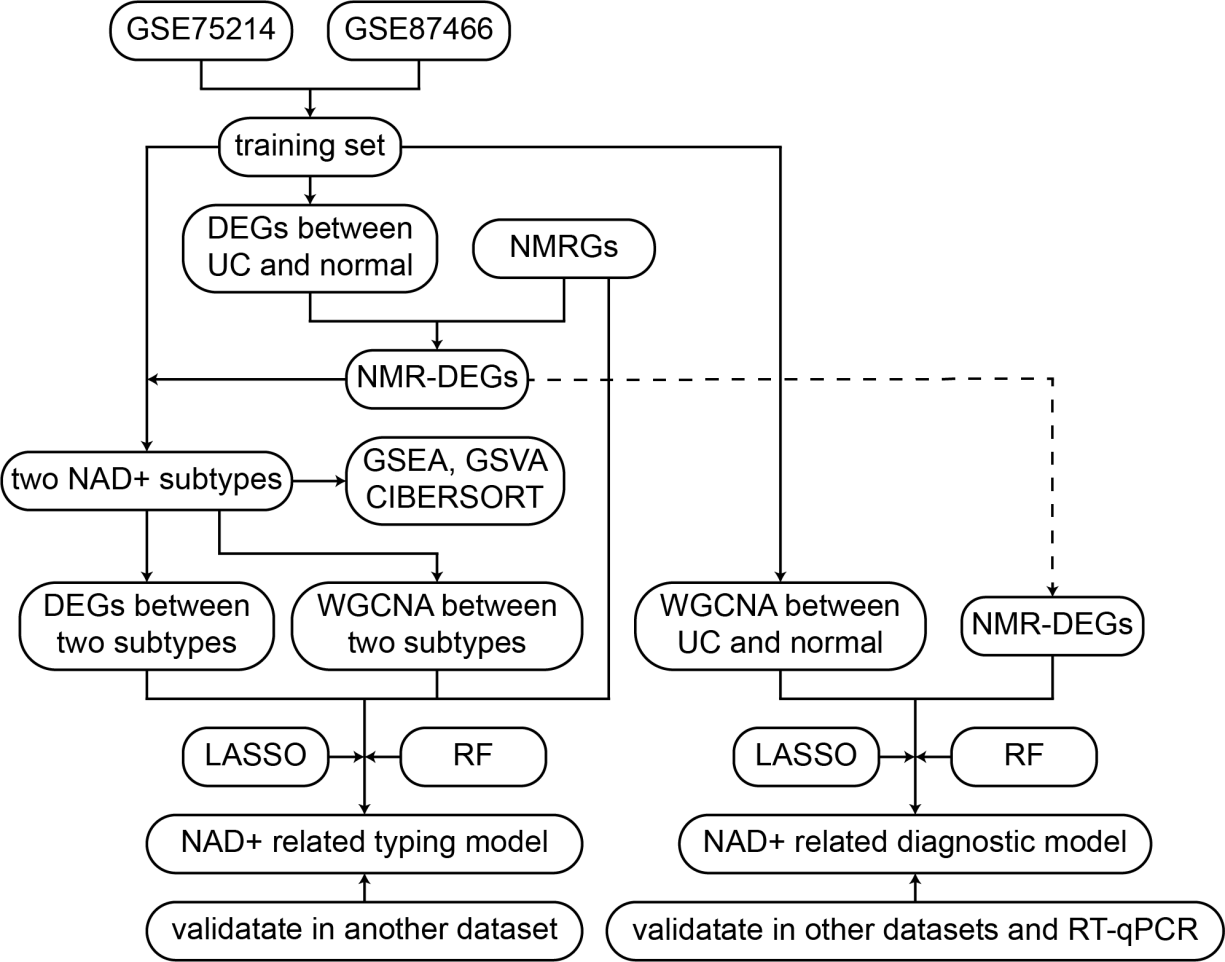
Flowchart of the research.

Herein, the UC datasets were sourced from the Gene Expression Omnibus (GEO) database, as presented in the flowchart in **Figure 1**. The training sets utilized GSE75214 and GSE87466, comprising 161 UC samples and 32 normal tissue samples (33, 34). GSE75214 contained a total of 97 UC samples, of which 74 were active UC samples and 23 were inactive UC samples. To ensure the accuracy of the analysis, we excluded the 23 inactive UC samples, and the final dataset used for analysis included 74 active UC patients. The SOFT files from these datasets were imported using the GEOquery package in R software (version 4.3.1). In order to merge gene expression data from multiple datasets, we first normalized and mapped the probe IDs to gene symbols. When multiple probes corresponded to a single gene, the avereps function from the limma package was used to compute average expression values. Subsequently, datasets were merged, and batch effects across datasets were adjusted using the ComBat function from the sva package. ComBat adjusts for systematic technical variations between datasets while retaining biological signal. Principal Component Analysis (PCA) was used to verify the effectiveness of the batch effect adjustment, which demonstrated successful removal of batch effects while maintaining the biological structure of the data. The external validation sets included GSE92415, GSE206285, and GSE66407 (35–38), which underwent the same preprocessing methods as the training set. **Supplementary Table 1** presents detailed information on all datasets.

### 2.2 Acquisition of NMRGs

NMRGs were curated from multiple databases, including the Kyoto Encyclopedia of Genes and Genomes (KEGG) pathway database (hsa00760), Reactome database (R-HSA-196807), and GeneCards database (NAD+ Metabolism Pathway). After removing duplicates, 54 NMRGs were identified. An intersection with all genes in the training set was performed, resulting in 47 NMRGs selected for subsequent analysis. **Supplementary Table 2** presents detailed information of these 47 NMRGs.

### 2.3 Identification and enrichment analysis of differentially expressed genes between UC and normal

Differential expression analysis between UC and normal samples in the training set was conducted using limma package (39), with significant differential expression defined by |log2 fold change (FC)| > 0.5 and false discovery rate (FDR) < 0.05. The resulting differentially expressed genes (DEGs) were visualized as volcano plots and heatmaps using the ggplot2 package. Enrichment analysis of these DEGs for Gene Ontology (GO) and KEGG pathways was performed using the clusterProfiler package, with gene annotation facilitated by the org.db package. Pathways with both p-values and q-values < 0.05 were considered significantly enriched.

### 2.4 Identification of NAD+ subtypes by DE-NMRGs

To focus on NAD+ metabolism-related genes, we intersected the DEGs between UC and normal with the list of NMRGs. This intersection resulted in the identification of DE-NMRGs, which were used for further analysis. We then performed unsupervised clustering on the UC samples using the ConsensusClusterPlus package based on the expression levels of these DE-NMRGs to identify subtypes of UC (40). The optimal number of clusters was determined by evaluating the cumulative distribution function, consistency clustering scores, and consensus clustering plots. Additionally, PCA was utilized to differentiate between the NAD+ subtypes. The boxplot and heatmap of these DE-NMRGs between subtypes were generated respectively using the ggpubr and pheatmap packages.

### 2.5 Identification and enrichment analysis of DEGs between NAD+ subtypes

Differential expression analysis between NAD+ subtypes was conducted using limma package, setting thresholds of |log2FC| > 0.5 and an adjusted p-value (FDR) < 0.05 to identify significant DEGs between NAD+ subtypes. Gene set enrichment analysis (GSEA) was performed with the clusterProfiler package (41, 42), and results were visualized using the enrichplot package. Gene set variation analysis (GSVA) was performed using the GSVA and GSEABase packages (43), with visualization facilitated by the pheatmap package. All gene sets were sourced from the molecular signature database (MSigDB). Gene sets with an FDR < 0.01 were considered statistically significant.

### 2.6 Immune cell infiltration analysis between NAD+ subtypes

The CIBERSORT package was used to analyze the abundance of 22 types of infiltrating immune cells in all samples (44), and the results were visualized using the ggplot2 package. Interactions among immune cells were examined using the corrplot package to further analyze the impact of immune cells in UC. Comparisons of relative immune cell abundances between normal samples and different NAD+ subtypes were visualized using the ggpubr package. Statistical comparisons were performed using the Wilcoxon rank-sum test, with P < 0.05 considered statistically significant.

### 2.7 Construction of co-expression networks in UC based on WGCNA

Co-expression networks were constructed from UC samples using the WGCNA package (45). Samples were subjected to hierarchical clustering to identify and remove outliers. The optimal soft-thresholding power was determined based on the scale-free topology fit index (R^2^ > 0.85). The gene expression matrix was transformed into a weighted adjacency matrix, which was subsequently converted into a topological overlap matrix (TOM). The TOM facilitated module detection via hierarchical clustering of the gene dendrogram, employing the dynamic tree-cutting method to identify modules and compute the module eigengenes, representing the principal components of gene expression profiles within each module. Correlations between each subtype and eigengenes of each module and the corresponding p-values were calculated to quantify the association of each module with different NAD+ subtypes. Gene significance scores within each module were computed to reflect their relative importance to different NAD+ subtypes.

### 2.8 Identification of key genes between NAD+ subtypes and construction of a classification model through machine learning

We initially intersected NMRGs, DEGs between NAD+ subtypes, and genes from WGCNA modules, resulting in several candidate NMRGs to identify key genes for differentiating NAD+ subtypes. Subsequently, we applied two machine learning techniques, LASSO and RF, to further select these NMRGs. LASSO regression, performed using the glmnet package in R, employs regularization to aid in feature selection, aiming to enhance the predictive accuracy and interpretability of the model. Additionally, RF analysis, conducted using the randomForest package in R, was selected for its high accuracy, sensitivity, and specificity, making it particularly suitable for handling biological data with complex interactions. The cross-validated genes identified were considered hub genes capable of effectively distinguishing between different NAD+ subtypes of UC. Scatter plots were generated using the regplot package to evaluate the typing efficacy of these genes, and ROC analysis was conducted with the pROC package to further validate the predictive performance of the model.

### 2.9 Construction of a diagnostic model for UC based on NMRGs

Similarly, we merged NMRGs, DEGs between UC and normal, and genes from WGCNA modules to identify several candidate NMRGs. These NMRGs were further refined using LASSO regression and RF analyses, with the cross-validated genes identified as hub genes capable of diagnosing UC. The construction of the co-expression network for this diagnostic model utilized the WGCNA package, but the scale-free topology fit index (R^2^) threshold was set at 0.80 to meet the specific needs of the diagnostic model. The constructed diagnostic model was validated using scatter plots and ROC analysis to assess its efficacy in UC diagnosis.

### 2.10 Cell culture and RT-qPCR

To validate whether gene expression changes identified in the diagnostic model could be confirmed experimentally, we used the normal human colonic epithelial cell line NCM460. The cells were divided into control and LPS-treated (10 μg/mL) groups. LPS stimulation was chosen to model the inflammatory environment characteristic of UC. LPS, a bacterial endotoxin, activates immune responses through Toll-like receptor 4 (TLR4), which plays a central role in UC pathogenesis by triggering inflammation and disrupting the epithelial barrier. LPS-induced inflammation in NCM460 cells mimics the epithelial cell response to microbial stimuli in UC (46, 47). Cells were cultured in RPMI 1640 medium supplemented with 10% fetal bovine serum at 37°C in a 5% CO_2_ atmosphere. After reaching the logarithmic growth phase, the treatment group cells were exposed to LPS for 24 h. RNA extraction was performed using TaKaRa’s RNAiso Plus (Trizol), and reverse transcription was conducted using TOYOBO’s ReverTra Ace^®^ qPCR RT Master Mix. Fluorescent quantitative PCR analysis was performed on the ABI 7900HT FAST system using Thermo’s Power SYBR Green PCR Master Mix. Experimental data were analyzed using GraphPad Prism 9.5.0 software, with statistical significance assessed using an unpaired *t*-test, and P < 0.05 considered statistically significant.

### 2.11 Statistical analysis

R software (version 4.3.1) was used for data analysis. The statistical significance of normally distributed continuous variables was assessed using independent student’s *t*-tests, while differences in non-normally distributed continuous variables were evaluated using the Wilcoxon rank-sum test. For multiple comparisons, the Benjamini–Hochberg method was applied to adjust the p-values and control the FDR. This method ranks the p-values from all tests and adjusts them based on their rank relative to the total number of tests, ensuring that the FDR is controlled. Furthermore, ROC analysis was used to evaluate the typing and diagnostic biomarkers and models. Spearman correlation analysis was employed to examine the relationships between infiltrating immune cells and gene biomarkers. All statistical tests were two-tailed, with significance levels set at P < 0.05. Significance results were indicated with asterisks: “ns” denotes P > 0.05, “*” denotes P < 0.05, “**” denotes P < 0.01, and “***” denotes P < 0.001.

## 3 Results

### 3.1 Identification of DEGs in UC

**Figure 2:**
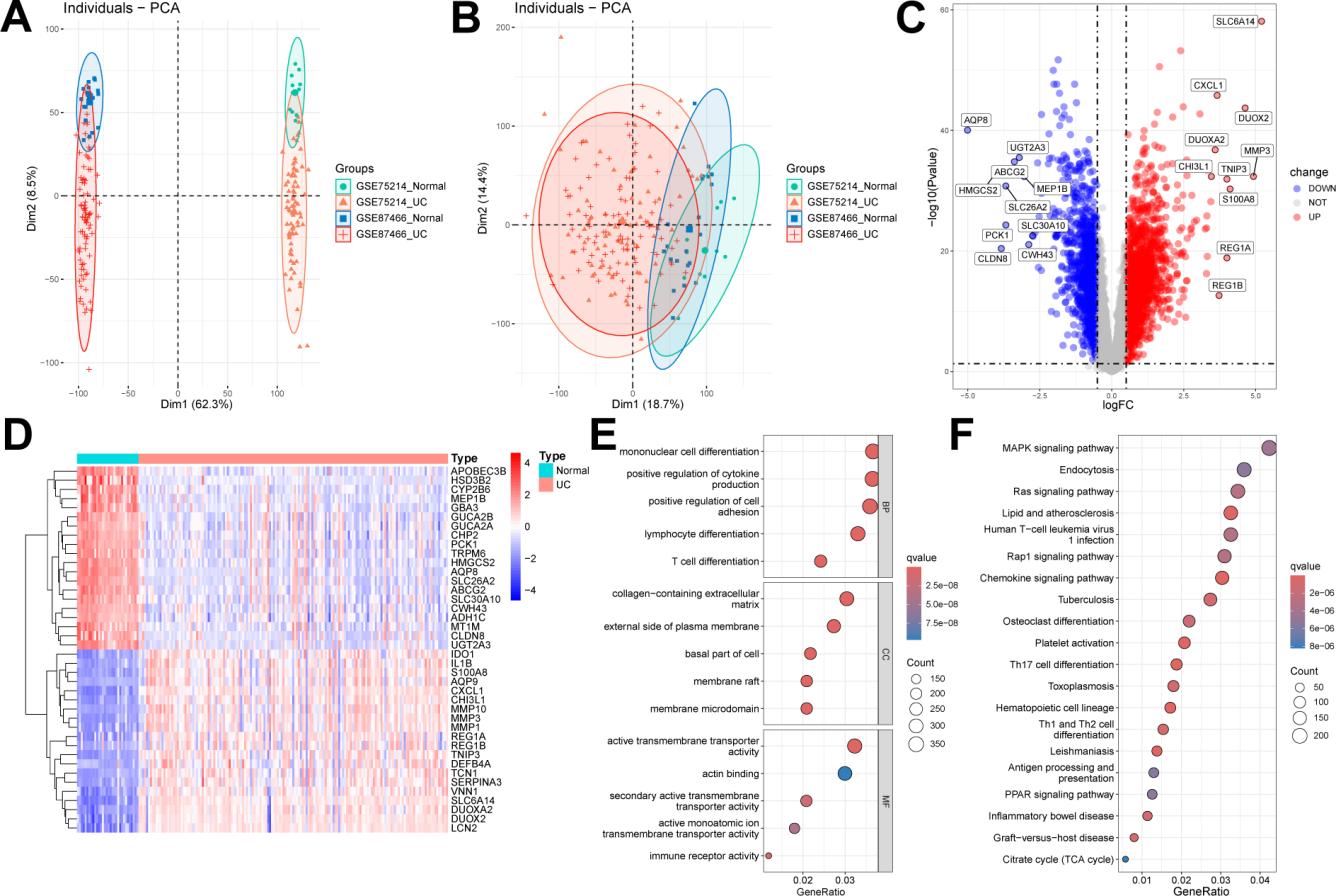
Identification and enrichment analysis of DEGs. **(A, B)** Two datasets (GSE75214, GSE87466) were combined into one dataset after removing batch effects. Sample relationships before and after batch effect removal. **(C, D)** Volcano plot and heatmap showing DEGs between UC samples and normal samples. **(E)** GO enrichment analysis results of DEGs. **(F)** KEGG enrichment analysis results of DEGs.

We selected and downloaded two human UC microarray datasets (GSE75214 and GSE87466) from the GEO online database. After careful screening, the study included 161 patients with UC and 32 control participants. Specifically, GSE75214 included 74 UC tissues in an active disease state and 11 normal colonic tissues, while GSE87466 included 87 UC tissues and 21 normal colonic tissues. After removing batch effects, the two datasets were merged into a UC training set, resulting in 17,348 genes. Samples from these independent datasets exhibited distinct clustering before batch effect removal (**Figure 2A**) but clustered together post-removal (**Figure 2B**). Subsequently, using the “limma” package in R with a threshold of FDR < 0.05 and |log2FC| > 0.5, we identified 2,935 DEGs, including 1,738 upregulated and 1,197 downregulated genes (**Figures 2C, D**).

### 3.2 Functional and pathway enrichment analysis of DEGs

We conducted GO and KEGG enrichment analyses on the 2,935 DEGs to gain deeper insights into their biological functions. The GO enrichment analysis revealed that DEGs were significantly enriched in multiple biological processes, cellular components, and molecular functions. Specifically, the enriched biological processes included mononuclear cell differentiation, positive regulation of cytokine production, and lymphocyte differentiation; the cellular components primarily involved the collagen-containing extracellular matrix and the external side of plasma membrane; and the molecular functions included active transmembrane transporter activity and actin binding (**Figure 2E**). In the KEGG enrichment analysis, genes were predominantly enriched in pathways, including the mitogen-activated protein kinase (MAPK) signaling pathway, endocytosis, and chemokine signaling pathways (**Figure 2F**).

### 3.3 Identification of two UC subtypes based on DE-NMRGs

**Figure 3:**
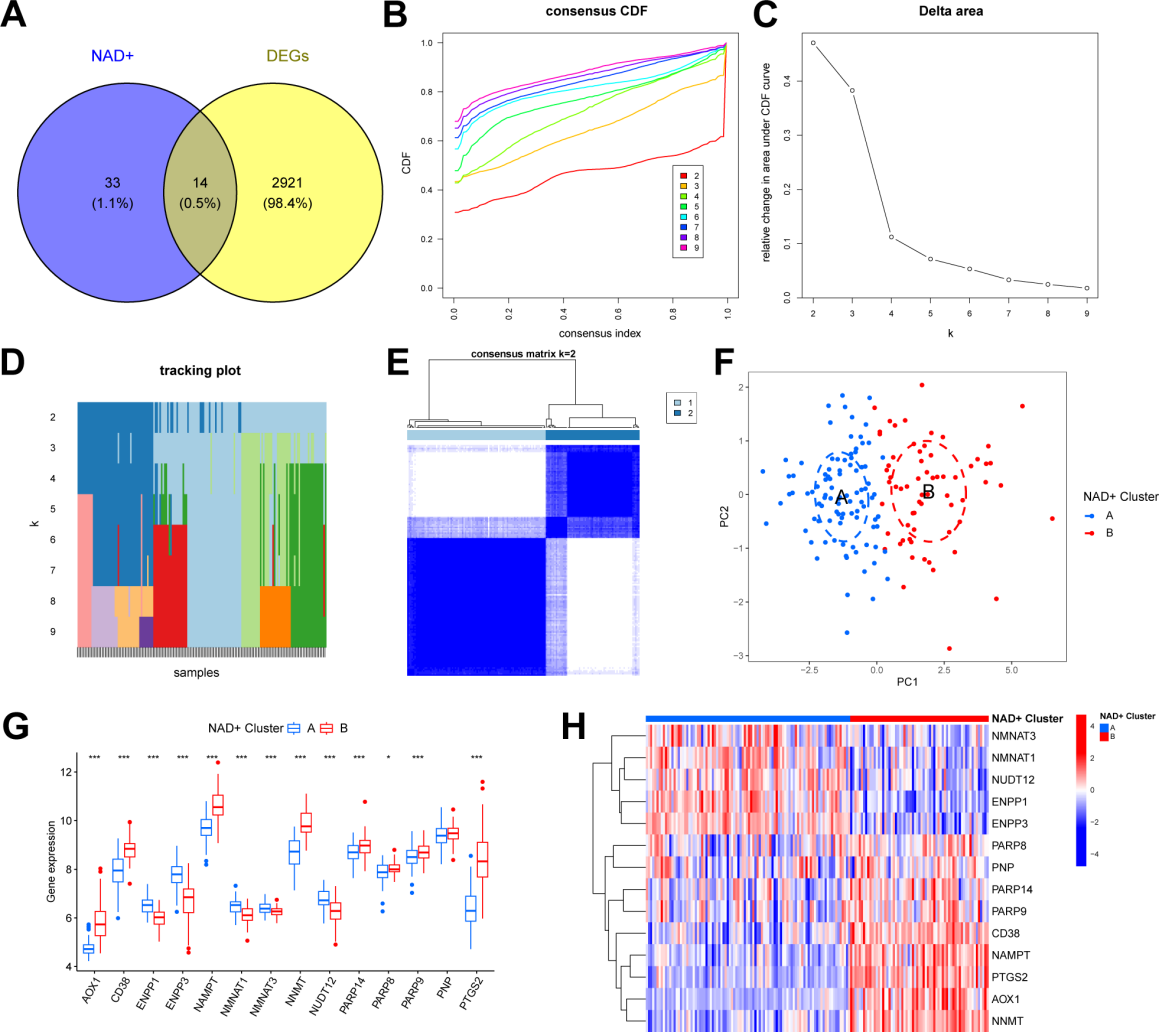
Identification of two NAD+ subtypes in UC. **(A)** Venn diagram showing the overlapping genes between DEGs and NMRGs. **(B)** Consensus cumulative distribution function (CDF) plot showing the area under the curve for k = 2-9. **(C)** Relative change in the area under the CDF curve. **(D)** Tracking plot showing the sample subtypes for different values of (k). **(E)** Consensus matrix heatmap for k = 2. **(F)** PCA plot showing the distribution of the two subtypes. **(G, H)** Boxplot **(G)** and heatmap **(H)** displaying the differential expression of DE-NMRGs between the two NAD+ subtypes. * p < 0.05; *** p < 0.001.

We intersected the identified DEGs with NMRGs to elucidate the NAD+ subtypes in UC and identified 14 NMRGs as DEGs between UC and normal samples (DE-NMRGs) (**Figure 3A**). Based on the expression profiles of these 14 DE-NMRGs, we performed consensus unsupervised clustering analysis on the 161 UC samples in the training set. The results indicated that, at k = 2, the patients with UC clustered into two subgroups with good internal consistency and stability (**Figures 3B–D**). Combined with the results from the consensus matrix heatmap (**Figure 3E**), we categorized the 161 UC samples into two subtypes: cluster A (n = 96) and cluster B (n = 65). Furthermore, PCA further confirmed the clear separation between these two subtypes (**Figure 3F**). The box plot and heatmap display the differential gene expression patterns of DE-NMRGs between subtypes (**Figures 3G, H**).

### 3.4 Functional enrichment analysis between NAD+ subtypes

**Figure 4:**
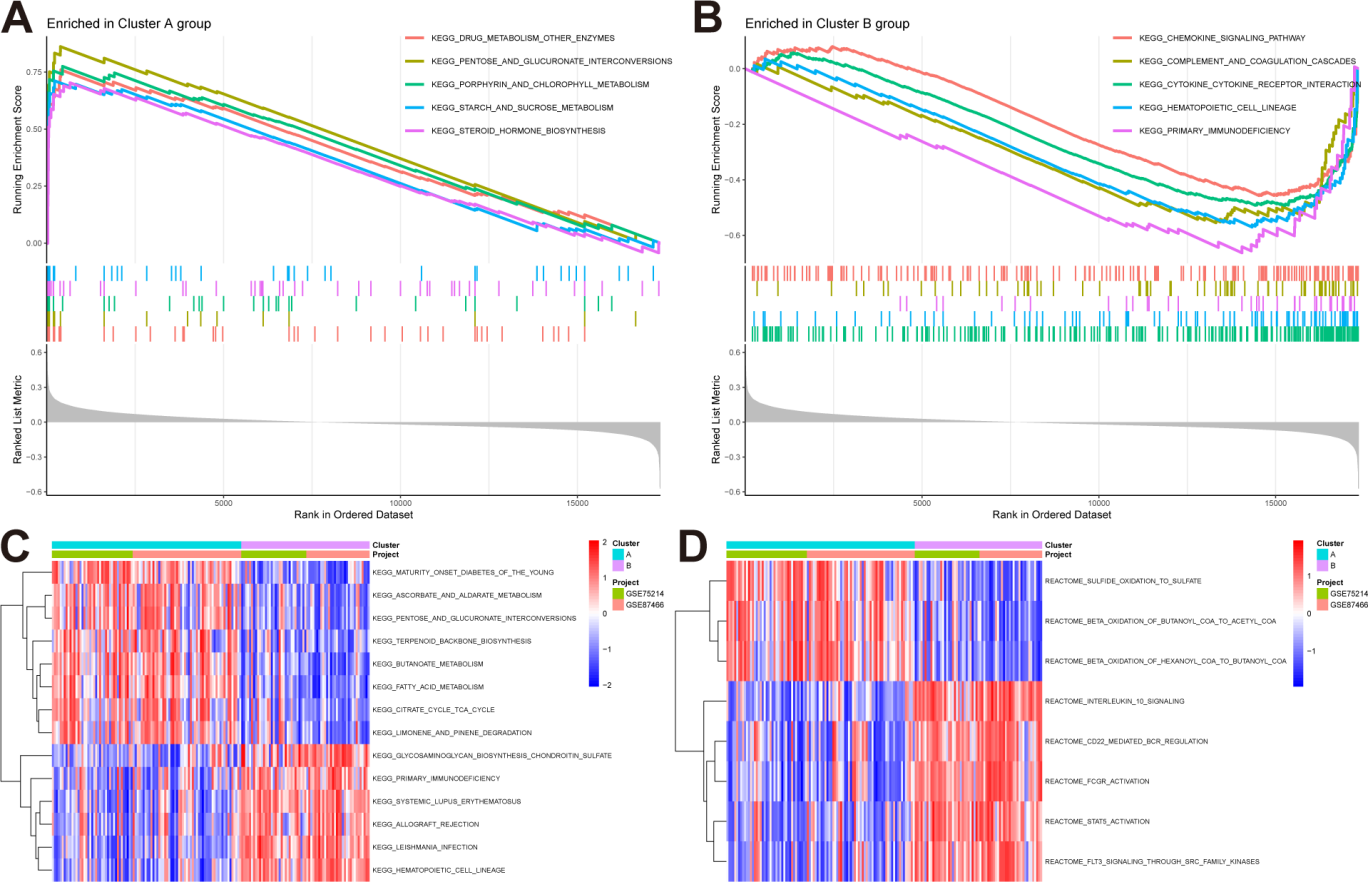
Pathway enrichment analysis reveals distinct biological behaviors of NAD+ subtypes in UC. **(A, B)** GSEA highlights pathways significantly enriched in subtype A and B. **(C, D)** GSVA result, **(C)** Enriched pathways based on KEGG pathways. **(D)** Enriched pathways based on Reactome pathways.

We conducted GSEA and GSVA enrichment analyses to explore the biological and behavioral differences between the two subtypes. In the GSEA analysis, the subtypes exhibited some distinctions: pathways including drug metabolism–other enzymes, and pentose and glucuronate interconversions were significantly enriched in subtype A (**Figure 4A**), while pathways such as the chemokine signaling pathway and complement and coagulation cascades were significantly enriched in subtype B (**Figure 4B**). Furthermore, we performed GSVA analysis to assess the differences in pathway activities and biological functions between the two subtypes. The results indicated that pathways including maturity–onset diabetes of the young and ascorbate and aldarate metabolism were upregulated in subtype A, whereas glycosaminoglycan biosynthesis-chondroitin sulfate, and primary immunodeficiency were upregulated in subtype B (**Figure 4C**). Additionally, based on the reactome pathways, subtype A was primarily involved in sulfide oxidation to sulfate and beta-oxidation of butanoyl-CoA to acetyl-CoA, while subtype B was primarily involved in interleukin-10 signaling and CD22-mediated BCR regulation (**Figure 4D**).

### 3.5 Assessment of immune cell infiltration between NAD+ subtypes

**Figure 5:**
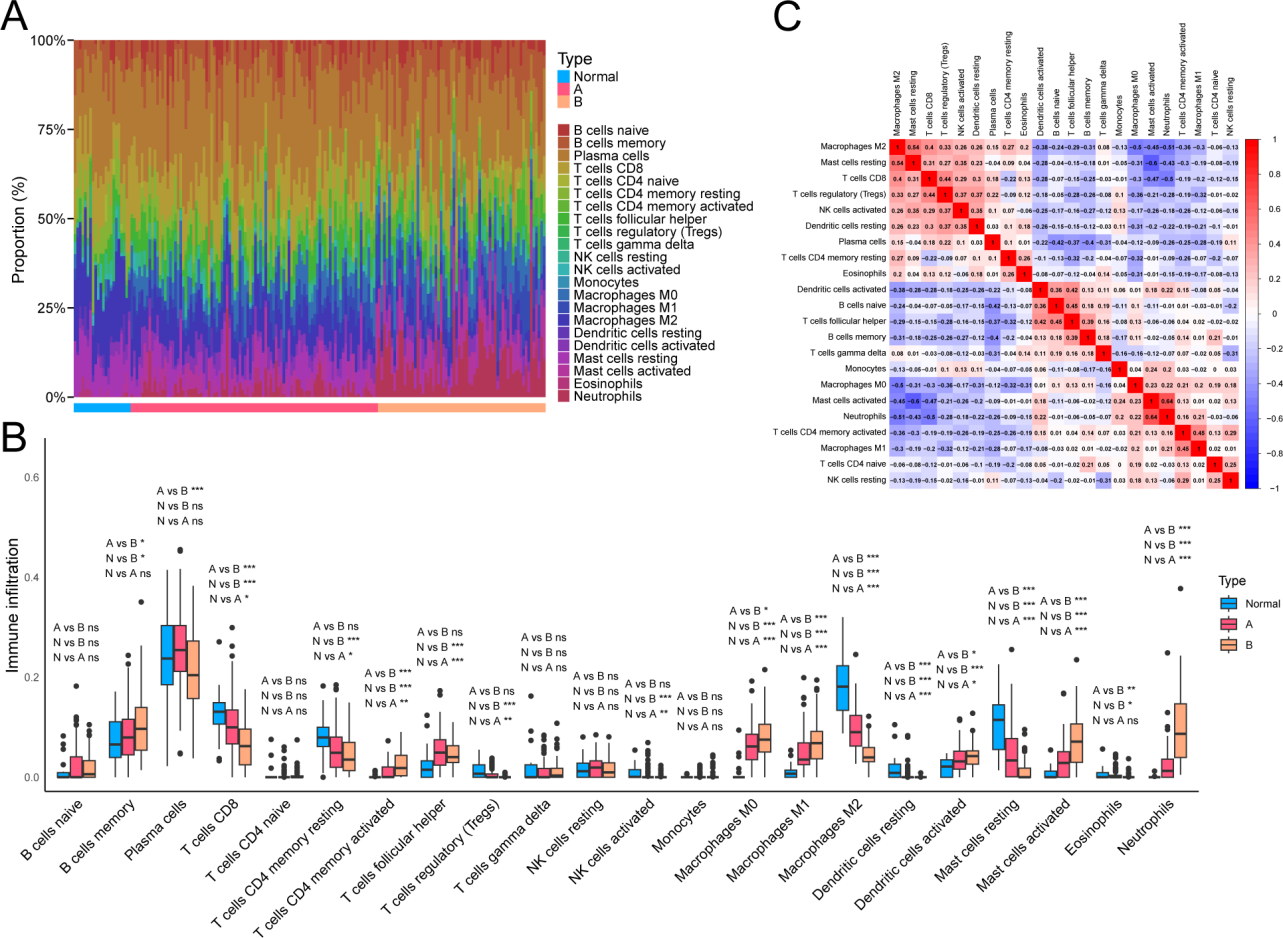
Immune cell infiltration profiles related to NAD+ subtypes in UC. **(A)** Heatmap showing the relative abundance of 22 immune cell types in different NAD+ subtype samples. **(B)** Boxplot visualizing the distribution and variability of immune cell relative abundance in NAD+ subtypes. **(C)** Correlation matrix describing the interactions between different immune cells. ns, not significant; * p < 0.05; ** p < 0.01; *** p < 0.001.

We assessed the infiltration proportions of different immune cells in UC samples using CIBERSORT to explore further the potential molecular mechanisms through which molecular subtypes influence UC progression, thereby analyzing the relationship between different NAD+ subtypes and immune cell infiltration. We found that compared with subtype A, subtype B exhibited significantly lower expression levels of M2 macrophages and resting mast cells, while M0 macrophages, M1 macrophages, activated mast cells, and neutrophils exhibited significantly higher expression. Additionally, compared to normal tissue, UC exhibited higher expression of activated memory CD4^+^ T cells, follicular helper T cells, M0 macrophages, M1 macrophages, activated mast cells, and neutrophils, while CD8^+^ T cells, resting memory CD4^+^ T cells, M2 macrophages, and resting mast cells exhibited lower expression in UC (**Figures 5A, B**). Consequently, the immune cell infiltration pattern of subtype A appears to be intermediate between subtype B and normal tissue. Furthermore, a negative correlation was observed between neutrophils and M2 macrophages (r = –0.51) and a positive correlation between neutrophils and activated mast cells (r = 0.64), and between resting mast cells and M2 macrophages (r = 0.54) (**Figure 5C**).

### 3.6 Differential analysis and WGCNA analysis between NAD+ subtypes

**Figure 6:**
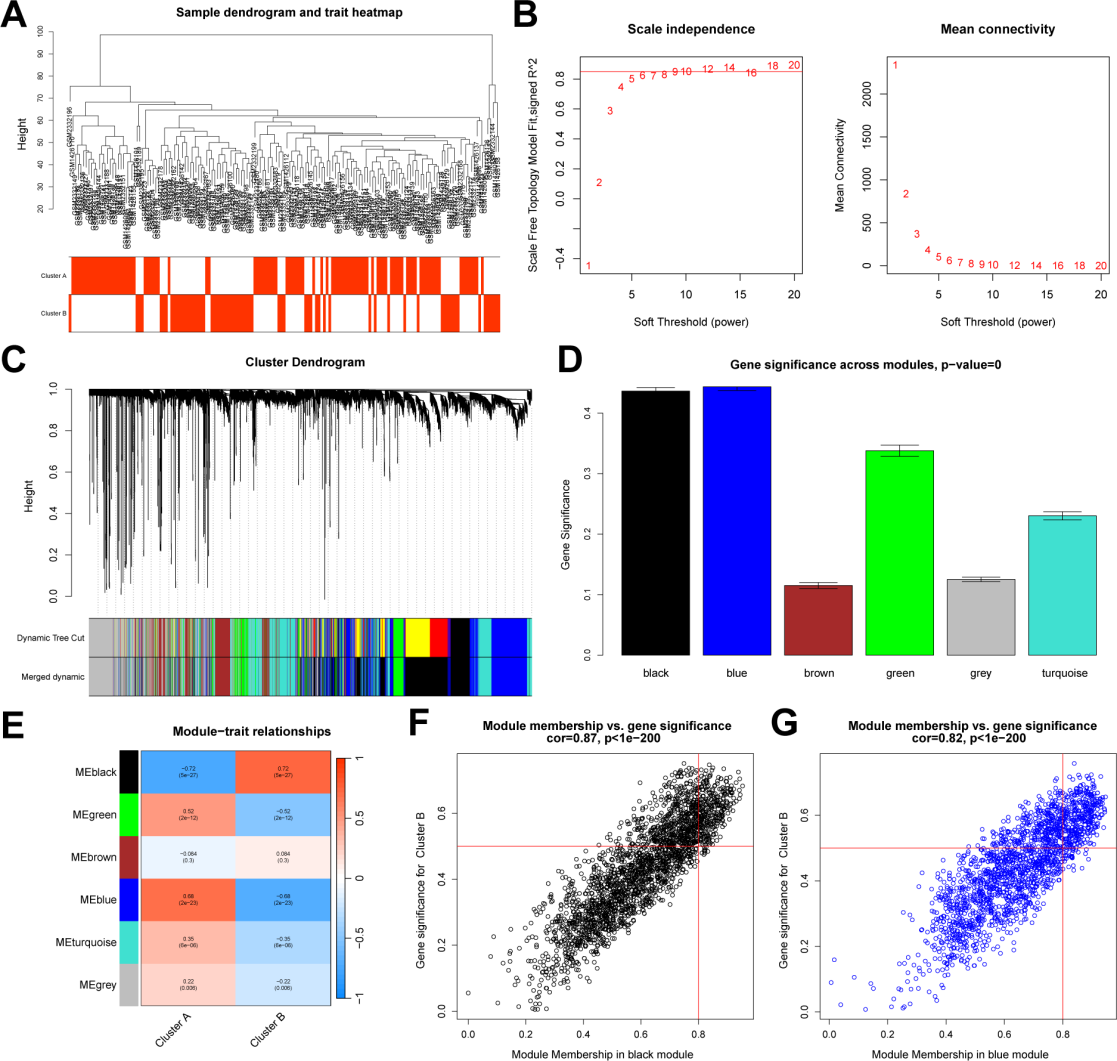
WGCNA between NAD+ subtypes. **(A)** Sample dendrogram generated after clustering using Pearson correlation coefficients and removal of outliers. **(B)** Determination of the soft-thresholding power in WGCNA. **(C)** Dendrogram of all DEGs between subtypes, clustered based on differential measurements, dividing genes into six different modules, each representing a co-expressed gene cluster. **(D)** Bar graph illustrating the significance measurements of the identified gene modules. **(E)** Heatmap of the UC module feature genes and their correlations with different NAD+ subtypes. **(F, G)** Scatter plots demonstrating the relationship between module membership and gene significance within the black and blue modules.

We conducted a differential analysis between the two subtypes and identified 1,597 DEGs. Subsequently, based on the entire gene expression profile, we applied the WGCNA algorithm to construct a co-expression network and key modules most correlated with the NAD+ subtypes. We used Pearson correlation coefficients to cluster the samples, and after removing outliers, we plotted a sample clustering dendrogram (**Figure 6A**). The optimal soft-thresholding power was set to 10 to maintain a scale-free topology and high connectivity (**Figure 6B**). Using hierarchical clustering, the clustering tree was divided and merged into six modules with different colors (**Figures 6C, D**). Among these modules, the black module (containing 2,386 genes) exhibited the highest correlation with subtype B (R = 0.72), and the blue module (containing 1,852 genes) was most correlated with subtype A (R = 0.68) (**Figure 6E**). Additionally, module membership in black module and its genes significance exhibited a significant correlation (cor = 0.87) (**Figure 6F**), and the blue module exhibited a correlation of cor = 0.82 (**Figure 6G**). Therefore, the black and blue modules were selected for further analysis.

### 3.7 Construction and validation of an NAD+ related typing model

**Figure 7:**
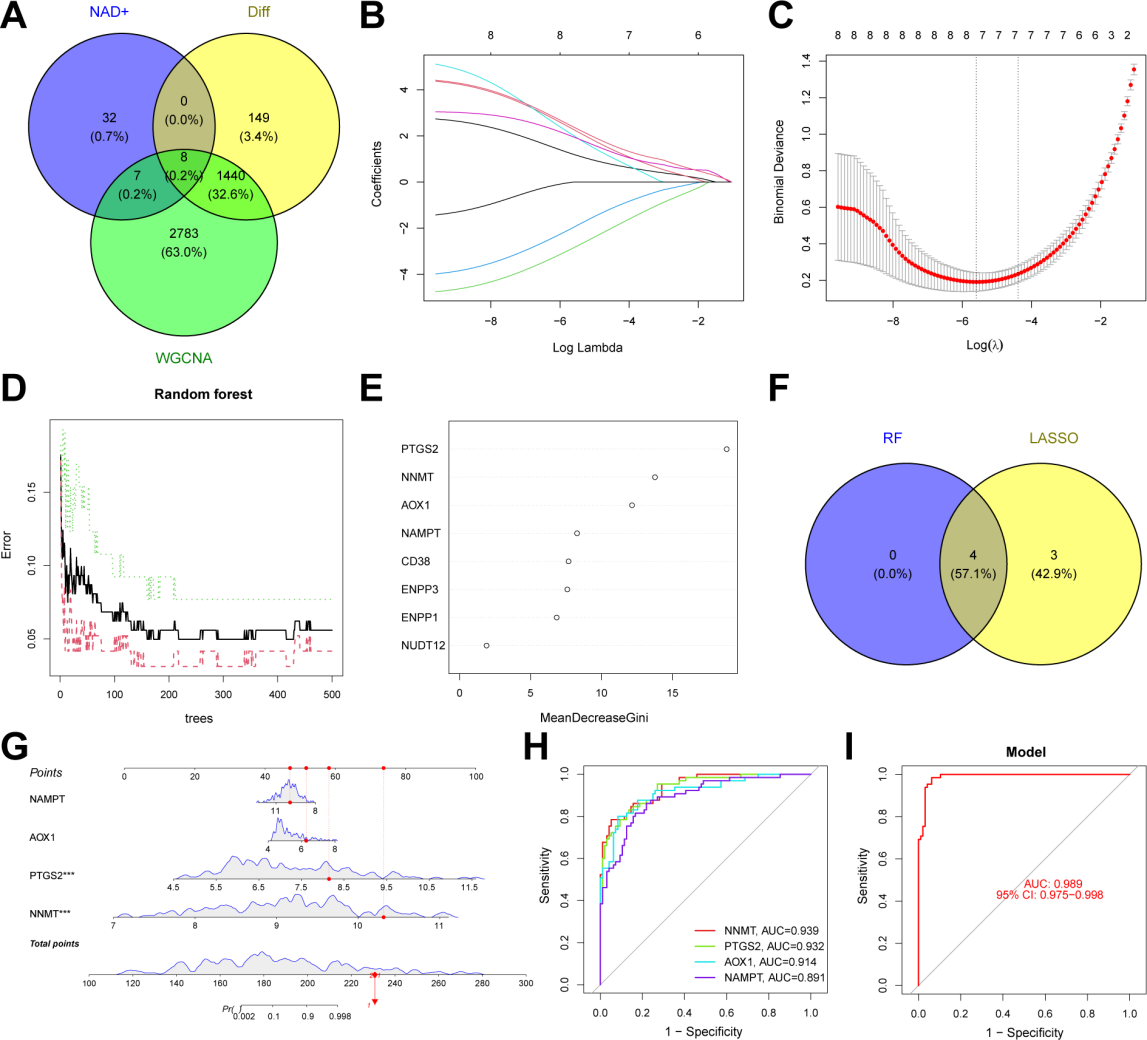
Construction of an NAD+ related typing model. **(A)** Venn diagram showing the intersection of DEGs between subtypes, key module genes identified by the WGCNA algorithm, and NMRGs, resulting in eight intersecting genes. **(B, C)** Feature gene selection using LASSO regression. **(D, E)** Feature gene selection using RF algorithms. **(F)** Venn diagram displaying four candidate hub genes identified by the aforementioned machine learning algorithms as the core of the predictive model. **(G)** Nomogram of the NAD+ related typing model in the training set. **(H, I)** The ROC curves of the four hub genes (AOX1, NAMPT, NNMT, and PTGS2) and the nomogram in the training set.

We constructed an NAD+-related predictive model to further clarify the role of NAD+ genes in the heterogeneity of patients with UC. Initially, we intersected the 1,597 DEGs between NAD+ subtypes, key module genes identified using the WGCNA algorithm, and all NMRGs, yielding eight intersecting genes (**Figure 7A**). Subsequently, we fitted the expression profiles of these eight intersecting genes into a LASSO regression analysis, determined the optimal value of λ, and selected seven potential key genes with non-zero coefficients in the training set (**Figures 7B, C**). Additionally, we implemented the RF algorithm in the training set and identified four effective predictive factors (**Figures 7D–E**). We identified four hub genes by merging the genes selected by these machine learning algorithms (**Figure 7F**): AOX1, NAMPT, NNMT, and PTGS2. We constructed a nomogram in the training set (**Figure 7G**) using these four hub genes and established ROC curves to evaluate the classification performance of each gene and the nomogram (**Figures 7H, I**) and found that the AUC for these hub genes were as follows: AOX1 (AUC = 0.914), NAMPT (AUC = 0.891), NNMT (AUC = 0.939), and PTGS2 (AUC = 0.932), with the nomogram achieving an AUC of 0.989. These results all indicate the accuracy of this model in predicting the NAD+ subtypes of UC.

**Figure 8:**
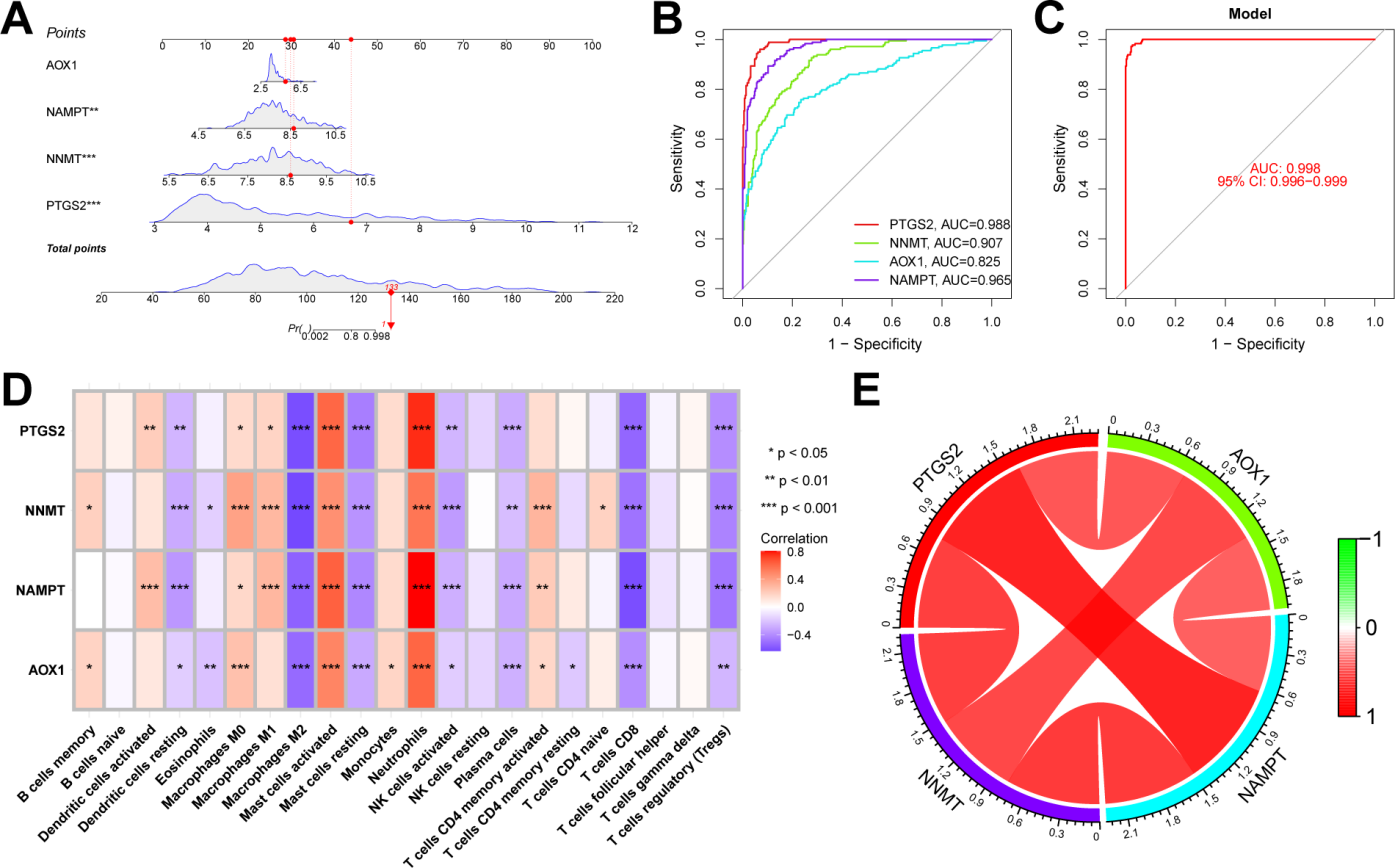
Validation of the NAD+ related typing model. **(A)** Nomogram of NAD+ related typing model in the validation set. **(B, C)** ROC curves for the four hub genes (AOX1, NAMPT, NNMT, and PTGS2) and the nomogram in the validation set. **(D)** Heatmap of the Spearman correlation coefficients between the expression of the four hub genes and the content of various immune cells. *p < 0.05; **p < 0.01; ***p < 0.001. **(E)** Network diagram illustrating the interrelationships among the four hub genes.

We conducted further external validation of these hub genes. We accessed a UC dataset from the GEO online database, GSE206285, and constructed a nomogram in this validation set based on these four hub genes (**Figure 8A**). We established ROC curves to evaluate the classification performance of each gene and the nomogram. The results revealed that in the validation set, the AUC for these hub genes were as follows: AOX1 (AUC = 0.825), NAMPT (AUC = 0.965), NNMT (AUC = 0.907), and PTGS2 (AUC = 0.988), with the nomogram achieving an AUC of 0.998. These results demonstrate the accuracy of these four key genes in predicting the NAD+ subtypes of UC (**Figures 8B, C**). Additionally, we evaluated the impact of these four hub genes on immune infiltration and conducted Spearman’s correlation analyses between gene expression levels and immune cell content. The results indicated that all four key genes strongly impacted immune cells (**Figure 8D**), and a strong correlation was observed among these four hub genes (**Figure 8E**).

### 3.8 Construction and validation of an NAD+ related diagnostic model in UC

**Figure 9:**
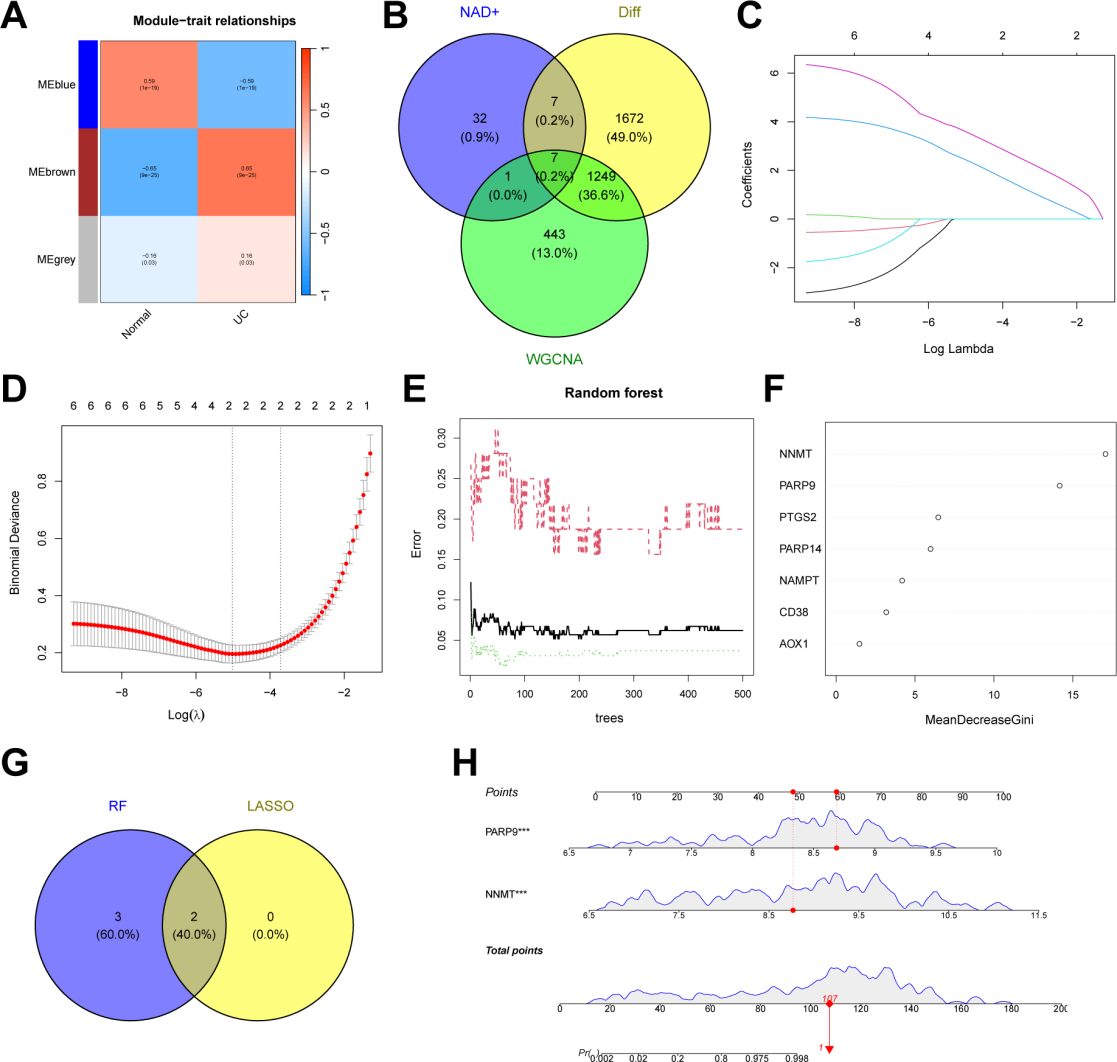
Construction of an NAD+ related diagnostic model in UC. **(A)** Heatmap of the UC module feature genes and their correlations with UC and normal in WGCNA. **(B)** Venn diagram showing the intersection of DEGs between UC and normal, key module genes identified by the WGCNA algorithm, and NMRGs, resulting in seven intersecting genes. **(C, D)** Feature gene selection using LASSO regression. **(E, F)** Feature gene selection using RF algorithms. **(G)** Venn diagram displaying two candidate hub genes identified by the aforementioned machine learning algorithms as the core of the predictive model. **(H)** Nomogram of NAD+ related diagnostic model in the training set.

**Figure 10:**
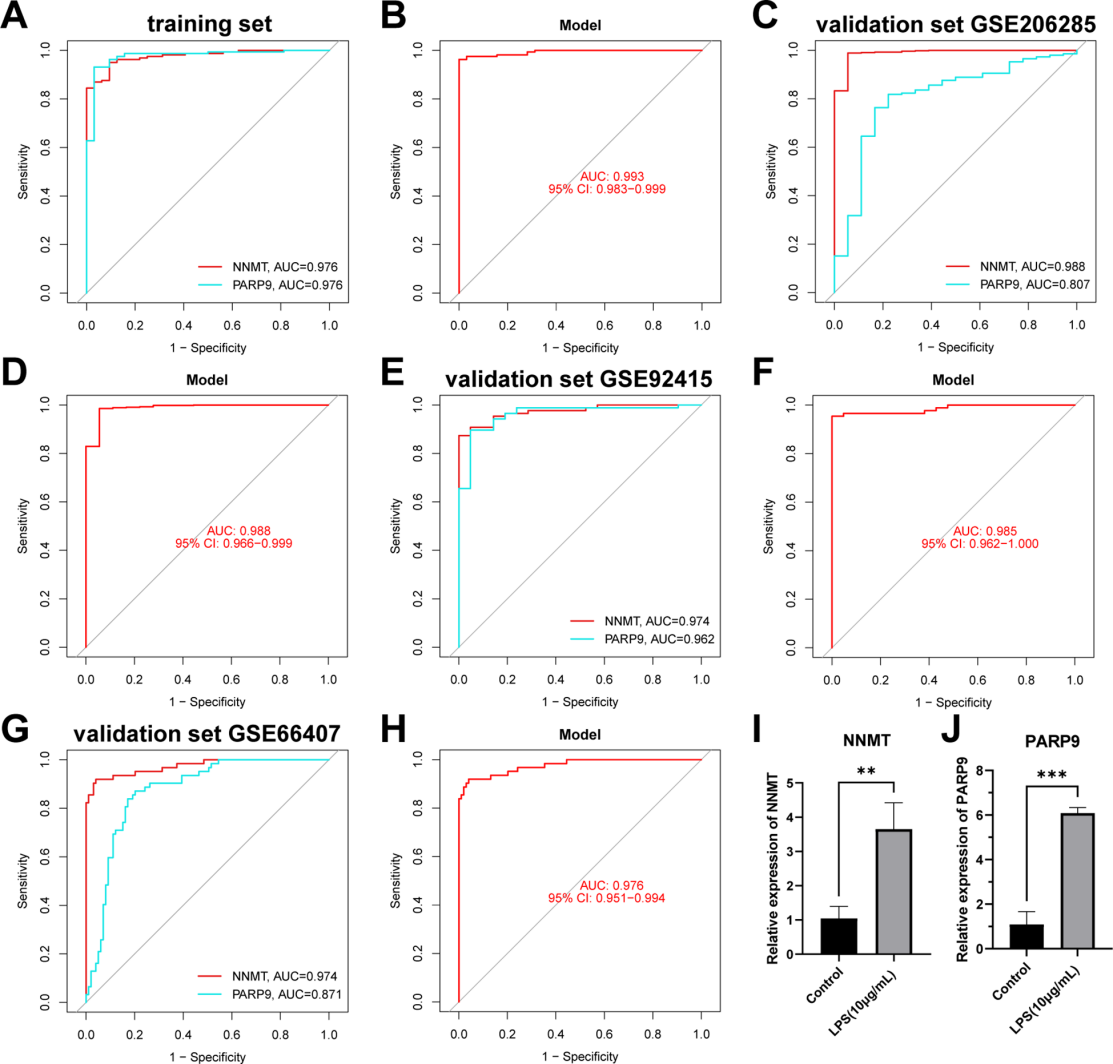
Validation of the NAD+ related diagnostic model in UC. **(A, B)** ROC curves for the two hub genes (NNMT and PARP9) and the nomogram in the training set. **(C–H)** ROC curves for the two hub genes (NNMT and PARP9) and the nomograms in the validation sets. **(I, J)** RT-qPCR experiment results of two hub genes (NNMT and PARP9) in NAD+ related diagnostic model. **p < 0.01; ***p< 0.001.

In addition to the typing model, we established a diagnostic model related to NAD+ metabolism genes in UC to clarify further the role of NAD+ genes in predicting UC. Initially, we constructed a co-expression network and key modules most correlated with UC and normal samples using the WGCNA algorithm based on the entire gene expression profile, selecting the brown module (R = 0.65) for further analysis (**Figure 9A**, **Supplementary Figure 1**). We intersected the DEGs identified between UC and normal tissues with the WGCNA brown module, obtaining seven intersecting genes (AOX1, CD38, NAMPT, NNMT, PTGS2, PARP14, and PARP9) (**Figure 9B**). Subsequently, we further filtered these genes using LASSO regression (**Figures 9C, D**) and RF (**Figures 9E, F**), merging the genes selected by machine learning algorithms and identifying two hub genes (**Figure 9G**). A nomogram was constructed based on these two hub genes in the training set (**Figures 9H**), and ROC curves were established to evaluate the classification performance of each gene and the nomogram (**Figures 10A, B**). The results demonstrated that the AUCs for these hub genes were NNMT (AUC = 0.976) and PARP 9 (AUC = 0.976), with the nomogram achieving an AUC of 0.993. These results indicate the accuracy of this model in predicting UC.

We conducted further external validation of the model using three UC GEO datasets (GSE206285, GSE92415, and GSE66407) from the GEO online database. Nomograms was constructed based on the two hub genes in the validation sets (**Supplementary Figure 2**), and ROC curves were established to evaluate the classification performance of each gene and the nomogram (**Figures 10C–H**). The results revealed that in the validation set GSE206285, the AUC for these hub genes was NNMT (AUC = 0.988) and PARP9 (AUC = 0.807), with the nomogram achieving an AUC of 0.988 (**Figures 10C, D**). In the validation set GSE92415, the AUC values for these hub genes were NNMT (AUC = 0.974) and PARP9 (AUC = 0.962), with the nomogram achieving an AUC of 0.985 (**Figures 10E, F**). In the validation set GSE66407, the AUC values for these hub genes were NNMT (AUC = 0.974) and PARP9 (AUC = 0.871), with the nomogram achieving an AUC of 0.976 (**Figures 10G–H**). These results all confirm the accuracy of these two key genes in the diagnosis of UC.

Subsequently, we conducted RT-qPCR experiments on the two hub genes, NNMT and PARP9, to further confirm their roles. The results revealed that NNMT and PARP9 expression was significantly increased in colonic epithelial cells following LPS treatment compared to the control group (**Figures 10I, J**). These RT-PCR results further corroborated the differential expression observed in the dataset analysis, highlighting the potential of these genes as biomarkers in the diagnosis of UC.

## 4 Discussion

UC is a chronic IBD that significantly affects the health and quality of life of patients. As a major global public health concern, the complex etiology and recurrent nature of UC make the research on effective diagnostic and personalized treatment approaches critical. Previous studies have identified the NAD+ metabolism as a critical pathway in UC pathogenesis. We categorized 161 UC samples collected from public databases into two distinct NAD+ subtypes, clusters A and B, to further explore the disease mechanisms. Cluster A exhibited stronger metabolic regulation and self-repair capabilities, while Cluster B was associated with more intense immune responses and severe tissue damage. Additionally, we demonstrated that NNMT and PARP9 are effective diagnostic biomarkers in UC, while AOX1, NAMPT, NNMT, and PTGS2 are discriminative markers for UC subtyping. Models developed using these biomarkers can predict disease progression more accurately and optimize treatment plans.

Following the GO and KEGG enrichment analyses of DEGs between UC samples and normal controls, we further validated the characteristics of UC as an immune-mediated inflammatory disease. The GO enrichment analysis revealed the central role of immune system regulation in UC, particularly the differentiation and function of monocytes and lymphocytes. Previous studies have suggested that the aberrant activity of these cells in UC may exacerbate the condition by promoting the release of inflammatory mediators and modulating the function of immune cells, leading to persistent tissue damage (48–51). Moreover, the excessive production of cytokines contributes to ongoing tissue injury (6, 52). KEGG enrichment analysis corroborated the GO findings, revealing the activation of critical pathways, including the MAPK and chemokine signaling pathways. Previous studies have reported that the MAPK signaling pathway is closely associated with UC progression (53, 54), with several therapeutic drugs alleviating UC symptoms by modulating this pathway (55, 56). Activation of the chemokine signaling pathway facilitates the migration of immune cells, including neutrophils and regulatory T cells, to inflamed areas, sustaining the inflammatory response and propelling disease progression (36, 57, 58). This was further validated in our subsequent analyses of immune cell infiltration. Elevated activated memory CD4^+^ T cells, follicular helper T cells, M0 macrophages, M1 macrophages, activated mast cells, and neutrophil expression in UC tissues highlight the significant increase in inflammation and immune activation in UC (59, 60). Conversely, lower expressions of resting memory CD4^+^ T cells, M2 macrophages, and resting mast cells in UC may reflect the limited functionality of these regulatory and reparative cells in the disease (61). These analyses confirm the significance of UC as an immune-mediated inflammatory disease and provide potential targets for future therapeutic interventions.

We observed that pathways related to metabolism and biosynthesis, including drug metabolism, carbohydrate conversion, and steroid hormone biosynthesis, were predominantly enriched in cluster A by comparing enriched pathways between the two UC subtypes. This suggests that cluster A possesses enhanced metabolic regulation and self-repair capabilities, which may help control inflammation spread, alleviate tissue damage, and promote damaged tissue regeneration (62–64). Conversely, pathways related to immunity and inflammation, including chemokine signaling, cytokine-cytokine receptor interaction, and complement and coagulation cascades, were significantly enriched in cluster B. This indicates that cluster B may be associated with more intense immune responses and severe tissue damage, where active inflammatory pathways could lead to rapid accumulation of immune cells and amplification of inflammatory reactions, complicating the disease progression and treatment (65, 66). Further analysis of immune cell infiltration between the subtypes supports these findings.

The infiltration levels of M0 and M1 macrophage, activated mast cells, and neutrophils are higher in subtype B than in subtype A. However, levels of M2 macrophage and resting mast cells are lower. This reflects a significantly different immune environment in subtype B compared with subtype A, potentially indicative of more intense inflammatory responses and reduced anti-inflammatory or tissue repair capabilities (67, 68). Additionally, we found that the pattern of immune cell infiltration in subtype A lies between that of subtype B and normal tissues, suggesting that subtype A may more closely resemble normal tissue compared to subtype B. These differences reveal fundamental distinctions between subtypes A and B based on immune response mechanisms, immune cell types and activity, and potential pathological processes. This highlights the importance of developing personalized treatment plans based on specific subtypes to optimize therapeutic outcomes and improve patient prognosis.

We propose that NNMT and PARP9 are effective diagnostic biomarkers for UC, while AOX1, NAMPT, NNMT, and PTGS2 can differentiate between UC subtype clusters A and B following comprehensive bioinformatics analysis and experimental validation.

Furthermore, NNMT (nicotinamide N-methyltransferase), a cytoplasmic enzyme primarily involved in the N-methylation of nicotinamide (Nam), reduces precursors of NAD+ through methylation and Nam excretion (69). Notably, NNMT helps maintain high levels of inflammatory signaling and sustained signal transduction by eliminating excess nicotinamide (24–27, 70). Consequently, elevated NNMT expression in UC may enhance the activation of inflammatory pathways and increase disease activity and tissue damage. Therefore, monitoring NNMT expression levels aids in diagnosing UC and in differentiating between disease activity states or subtypes, especially those related to inflammatory responses and metabolic status.

Besides, PARP(poly(ADP-ribose) polymerase, utilizing NAD+ as a substrate, facilitates ADP-ribosylation reactions during DNA damage (71), with increased PARP activity leading to decreased NAD+ levels (72). This process is crucial for DNA repair and regulating inflammatory responses. PARP9(poly(ADP-ribose) polymerase family member 9), a member of the PARP family, reveals expression patterns closely associated with immune responses and cellular stress states in various inflammatory diseases (30, 73–75). In UC, upregulated expression of PARP9 may relate to its role in cellular stress responses.

AOX1(aldehyde oxidase 1), a broad-spectrum oxidase, has demonstrated potential as a biomarker in various cancers, where its low expression in clear cell renal cell carcinoma and prostate cancer correlates with poor prognosis, suggesting its tumor-suppressing capabilities (76, 77). Although studies in UC are still limited, the role of AOX1 in regulating oxidative stress and inflammatory responses indicates its potential as a valuable target for personalized treatment in UC.

NAMPT(nicotinamide phosphoribosyltransferase) is critical in regulating the NAD+ pool and inflammatory responses in UC. It is a key enzyme in NAD+ biosynthesis and a cytokine, influencing cellular metabolism and immune responses within UC (20–23, 78). Elevated levels of NAMPT may reflect an adaptive response to metabolic demands and mucosal damage in UC (70, 79, 80), highlighting its potential as a biomarker for disease severity and subtype differentiation.

PTGS2 (prostaglandin-endoperoxide synthase 2, COX-2) is a crucial enzyme that converts arachidonic acid into prostaglandins, essential in inflammation and pain responses. In UC, significant upregulation of PTGS2 marks an inflammatory feature of the disease (81). Studies have reported that increased PTGS2 expression is closely associated with UC severity and progression, demonstrating its potential as a biomarker for disease activity and a therapeutic target (29, 82). Furthermore, the NAD+ metabolism pathway is closely linked to inflammatory response regulation, and changes in NAD+ metabolism may affect PTGS2 activity (28, 83), implying a potential interaction between NAD+ metabolism and PTGS2 in UC pathogenesis and management.

## 5 Conclusion

In conclusion, this study identified two UC subtypes associated with NAD+ metabolism, and our analysis of the differences between these subtypes highlighted the significant role of NAD+ metabolism in UC. We successfully identified key genes, including NNMT and PARP9, as diagnostic biomarkers for UC, and AOX1, NAMPT, NNMT, and PTGS2 for differentiating the NAD+ metabolism subtypes of UC. The nomograms developed from these biomarkers demonstrated exceptional accuracy and reliability in the early diagnosis and subtyping of UC, indicating the potential application of these biomarkers in UC treatment strategies. Future research should investigate the expression patterns of these genes in different patients with UC and their impact on treatment responses, which could help optimize treatment plans and advance therapeutic strategies for UC.

## Data Availability

Publicly available datasets were analyzed in this study. This data can be found here: https://www.ncbi.nlm.nih.gov/geo/.

## Supplementary material

Supplementary Figure 1 WGCNA between UC and normal samples. **(A)** Sample dendrogram generated after clustering using Pearson correlation coefficients and removal of outliers. **(B)** Determination of the soft-thresholding power in WGCNA. **(C)** Dendrogram of all DEGs between subtypes, clustered based on differential measurements, dividing genes into three different modules, each representing a co-expressed gene cluster. **(D)** Bar graph illustrating the significance measurements of the identified gene modules. **(E)** Scatter plots demonstrating the relationship between module membership and gene significance within the brown module.

Supplementary Figure 2 Nomograms of NAD+ related diagnostic module in validation sets. **(A–C)** Nomograms of NAD+ related diagnostic module in three validation sets.

Supplementary Table 1 The information of selected datasets in this study.

Supplementary Table 2 List of 47 NMRGs Selected for Subsequent Analysis.
