# Supplementary figures and images for: Interactions between NAD+ metabolism and immune cell infiltration in ulcerative colitis: subtype identification and development of novel diagnostic models
Source: Front Immunol. 2025 Feb 5;16:1479421. doi: 10.3389/fimmu.2025.1479421 (PMC11835821; doi:10.3389/fimmu.2025.1479421)

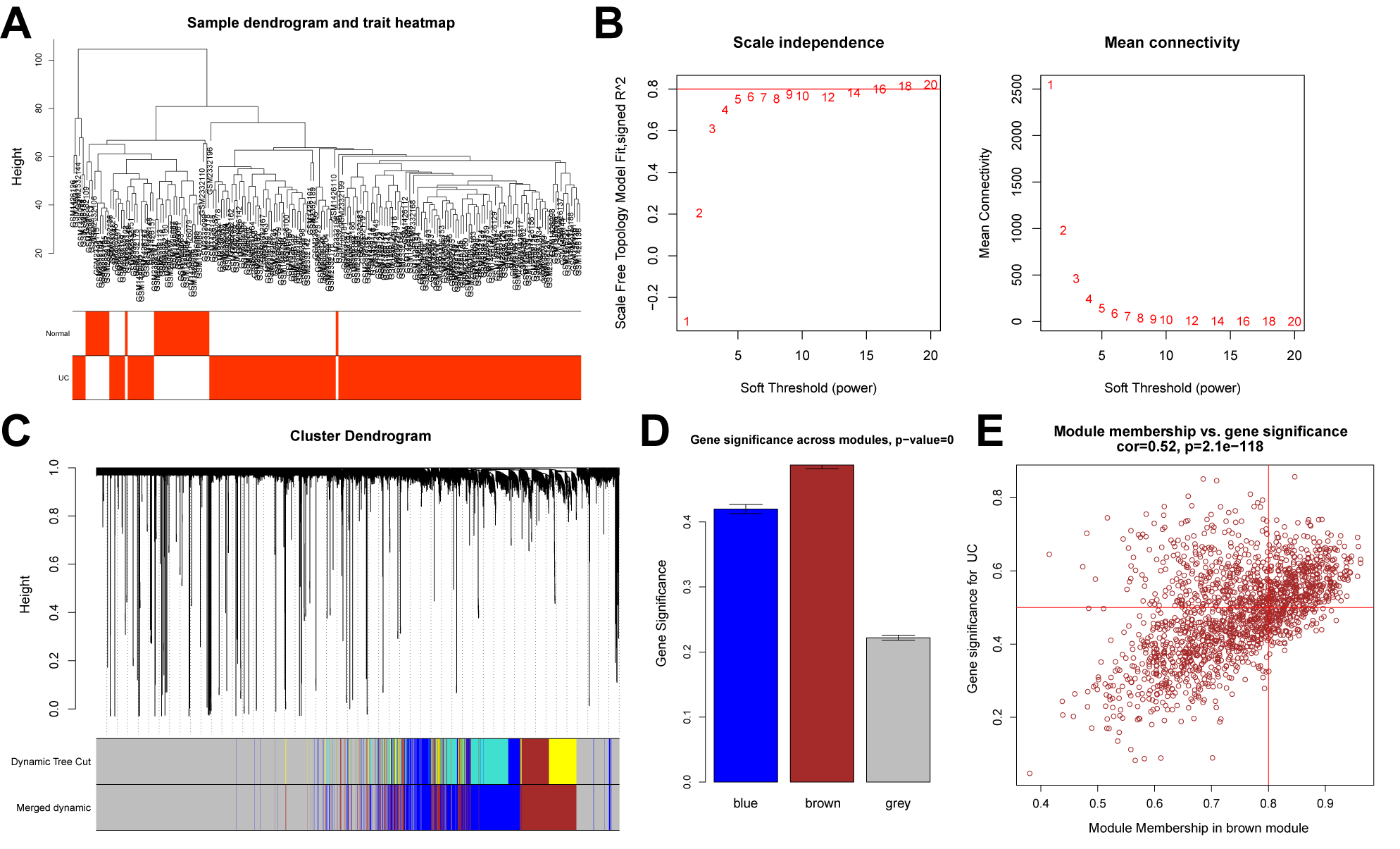

Supplement: Supplementary Figure 1 — WGCNA between UC and normal samples. (A) Sample dendrogram generated after clustering using Pearson correlation coefficients and removal of outliers. (B) Determination of the soft-thresholding power in WGCNA. (C) Dendrogram of all DEGs between subtypes, clustered based on differential measurements, dividing genes into three different modules, each representing a co-expressed gene cluster. (D) Bar graph illustrating the significance measurements of the identified gene modules. (E) Scatter plots demonstrating the relationship between module membership and gene significance within the brown module. [file Image1.tif]

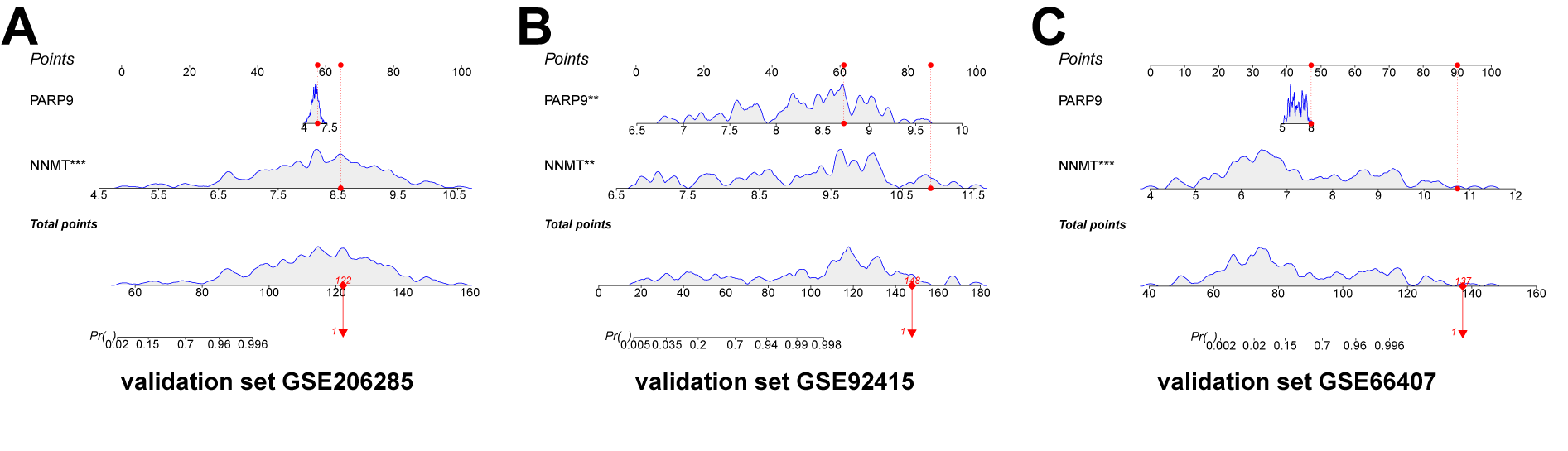

Supplement: Supplementary Figure 2 — Nomograms of NAD+ related diagnostic module in validation sets. (A–C) Nomograms of NAD+ related diagnostic module in three validation sets. [file Image2.tif]
